# Effect of Particle Size and Roasting Conditions on the Quality of High-Resistant Starch Rice

**DOI:** 10.3390/molecules31173076

**Published:** 2026-09-01

**Authors:** Jianhao Tang, Xingying Yu, Qi Zhao, Ruoyu Xiong, Jianjiang Bai, Ruifang Yang

**Affiliations:** Key Laboratory of Germplasm Innovation and Genetic Improvement of Grain and Oil Crops (Co-Construction by Ministry and Province), Ministry of Agriculture and Rural Affairs, Crop Breeding and Cultivation Research Institute, Shanghai Academy of Agricultural Sciences, Shanghai 201403, Chinayuxingying0327@163.com (X.Y.);

**Keywords:** high-resistant starch rice, roasting, grinding fineness, physicochemical properties, in vitro digestibility, glycemic index (GI)

## Abstract

This study investigated the effects of roasting temperature (130 °C, 150 °C, 180 °C) and grinding fineness (10, 50, and 100 mesh) on the nutritional quality and physicochemical properties of high-resistant starch rice flour (Youtangdao 3). The results revealed that the process of whole-grain moist roasting, followed by milling and sieving, markedly enhanced the resistant starch (RS) content while concurrently reducing the fraction of rapidly digestible starch (RDS). Specifically, roasting at 130 °C with coarse grinding (R130-10) yielded the highest absolute RS content (27.45 g/100 g). However, for fine-particle fractions essential for food texture, roasting at 150 °C (R150-100) proved optimal by achieving the highest RS proportion within the starch matrix (89.36% by Englyst method). Structural analysis via X-ray diffraction (XRD) and scanning electron microscopy (SEM) revealed that roasting facilitated the reorganization of starch chains. This molecular rearrangement led to a pronounced increase in crystallinity and induced distinct morphological alterations within the granules. Furthermore, Rapid Visco Analyzer (RVA) profiling indicated a substantial decline in peak viscosity for all roasted samples, indicating a significant loss of pasting ability and an increase in structural rigidity. In conclusion, the combination of moderate roasting temperature and precise particle size control effectively optimizes the nutritional value of high-RS rice flour. These insights provide a theoretical basis for developing high-RS rice flour ingredients.

## 1. Introduction

The rising prevalence of type 2 diabetes mellitus (T2DM) and related metabolic disorders has created an urgent demand for staple foods with reduced glycemic impact [1,2]. Among cereal-based staples, rice is of particular importance in Asian diets. However, conventional milled rice has a relatively high glycemic index (GI), prompting interest in processing strategies that reduce starch digestibility [3]. Resistant starch (RS), which escapes digestion in the small intestine and reaches the colon for fermentation, is a key determinant of low-GI properties in rice products. Starch is categorized into three types based on its digestion rate: rapidly digestible starch (RDS), slowly digestible starch (SDS), and resistant starch (RS) [4]. Rice (*Oryza sativa* L.), a staple food for much of the world’s population, is naturally high in RDS, resulting in a high postprandial glycemic index [5,6,7]. Therefore, modifying rice starch to boost its RS content while reducing RDS has emerged as a pivotal goal in contemporary food science research [5,8,9].

Physical modification techniques, particularly heat-moisture treatments (HMTs), have proven effective in altering starch functionality and digestibility [10,11,12,13]. Roasting, a traditional processing method utilizing dry heat via open flames or ovens, is typically associated with flavor development [14]. However, by precisely controlling the moisture content of the samples prior to roasting, this process can also be regarded as a form of HMT. In such cases, the internally retained moisture facilitates structural modifications during heating, thereby improving the physicochemical properties and functional characteristics of the starch. Furthermore, roasting induces starch gelatinization, dextrinization, and retrogradation, which collectively influence the formation of enzyme-resistant structures [15,16]. Previous studies have demonstrated that roasting can significantly increase RS content in various cereal grains by promoting the reassociation of amylose and amylopectin chains [16,17]. However, the structural transformations and digestibility profiles of starch are not determined by thermal history alone; particle size also plays a pivotal role, profoundly affecting the in vitro digestion of starch [18,19]. Mechanical grinding alters the specific surface area and physical integrity of starch granules, potentially affecting enzyme accessibility and hydrolysis kinetics [18]. While some studies suggest finer particles are more susceptible to digestion due to increased surface area [20], the interaction between particle size reduction and thermal modification in high-RS rice varieties remains underexplored.

In this context, Youtangdao 3, a naturally high-RS rice variety developed by the Shanghai Academy of Agricultural Sciences, provides a unique genetic background for studying starch modification [16]. Although this variety inherently contains higher levels of RS compared to conventional rice, its processing characteristics, particularly the response to roasting and grinding, require systematic investigation. The microstructure, crystallinity, and pasting properties of starch are highly sensitive to processing parameters such as temperature and particle size.

Therefore, this study aims to investigate the effects of roasting temperature (130 °C, 150 °C, and 180 °C) and grinding fineness (10-mesh, 50-mesh, and 100-mesh) on the physicochemical properties and in vitro digestibility of Youtangdao 3 rice flour. The study is designed in two tiers: (i) a broad screening of RS content across all temperature–mesh combinations to evaluate the combined effects of roasting and particle size on the primary outcome variable, and (ii) an in-depth structural and digestibility characterization (SEM, XRD, RVA, amylopectin chain-length distribution, and in vitro digestibility) focused on the 100-mesh fraction to isolate the thermal treatment effects on starch structure. These findings will provide a theoretical basis for optimizing processing parameters for high-RS rice flour applications.

## 2. Results

### 2.1. Effect of Roasting Conditions and Grinding Degree on RS Content

The effects of roasting temperature, duration, and grinding degree (mesh size) on the RS content of rice flour are presented in Table 1. Generally, roasting significantly enhanced the RS content compared to the untreated control groups (CK). The CK samples exhibited low RS levels, ranging from 11.39% to 13.87%. In contrast, thermal treatment markedly increased RS formation across all conditions. For instance, at 130 °C for 100 min, the RS content reached 27.45% (10-mesh). A similar trend was observed at higher temperatures; roasting at 150 °C for 70 min and 180 °C for 50 min resulted in peak RS contents of 25.30% and 24.24%, respectively, for the 10-mesh samples. Additionally, the degree of grinding influenced the RS content, with finer powders (100-mesh) generally showing slightly lower RS levels compared to coarser fractions (10-mesh) under the same roasting conditions.

Two-way ANOVA was performed to evaluate the individual and combined effects of roasting temperature and particle size on RS content. The results showed that both roasting temperature (F = 30.06, *p* < 0.001) and particle size (F = 15.15, *p* < 0.001) had highly significant main effects on RS formation, whereas their interaction was not significant (F = 0.90, *p* = 0.483), indicating that the effects of temperature and particle size on RS content were independent of each other. Post hoc Tukey’s HSD test revealed that roasting at 130 °C produced significantly higher RS content (26.55%) compared to 150 °C (23.90%) and 180 °C (23.17%), with no significant difference between the latter two temperatures. Regarding particle size, the 10-mesh fraction exhibited significantly higher RS content (25.67%) than the 100-mesh fraction (23.50%), while the 50-mesh fraction (24.43%) was not significantly different from either extreme.

### 2.2. Kinetics of RS Formation During High-Temperature Roasting

**Figure 1:**
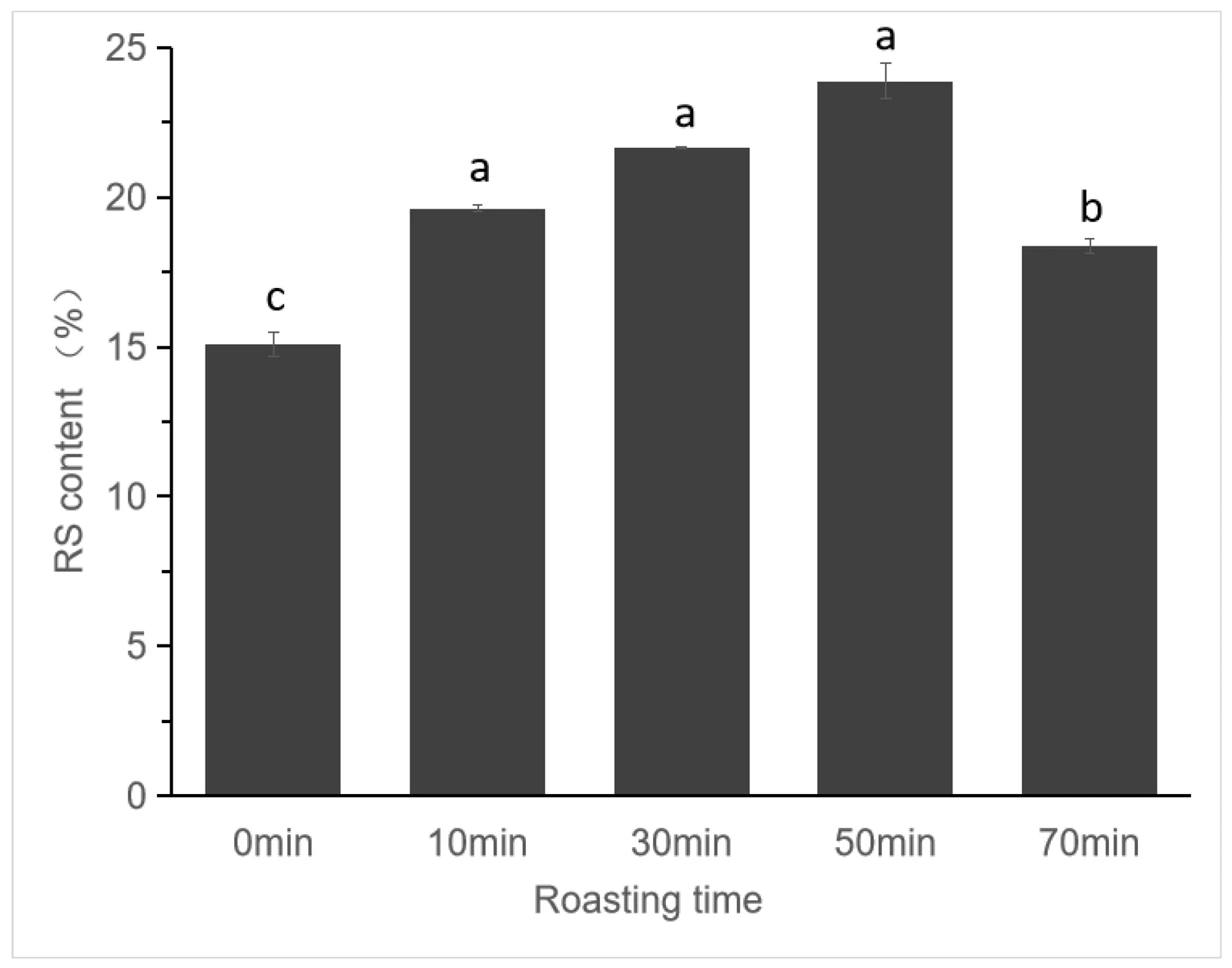
Effect of roasting time on the resistant starch (RS) content of rice flour. Rice samples were roasted at 180 °C for varying durations. Values represent the mean ± standard deviation. Different lowercase letters indicate significant differences among roasting times (*p* < 0.05).

To further explore the kinetic evolution during high-temperature processing, the RS content was tracked at 180 °C across varying time intervals. Measurements were taken at 0, 10, 30, 50, and 70 min to capture the distinct phases of the process (Figure 1). The results showed a non-linear trend where the RS content initially increased and then decreased. Starting from approximately 15.0% at 0 min, the RS content rose steadily, reaching a maximum value of nearly 24.0% at 50 min. However, extending the roasting time to 70 min caused a significant decline in RS content to about 18.5%, indicating that while moderate roasting promotes RS formation, excessive heating is detrimental.

**Figure 2:**
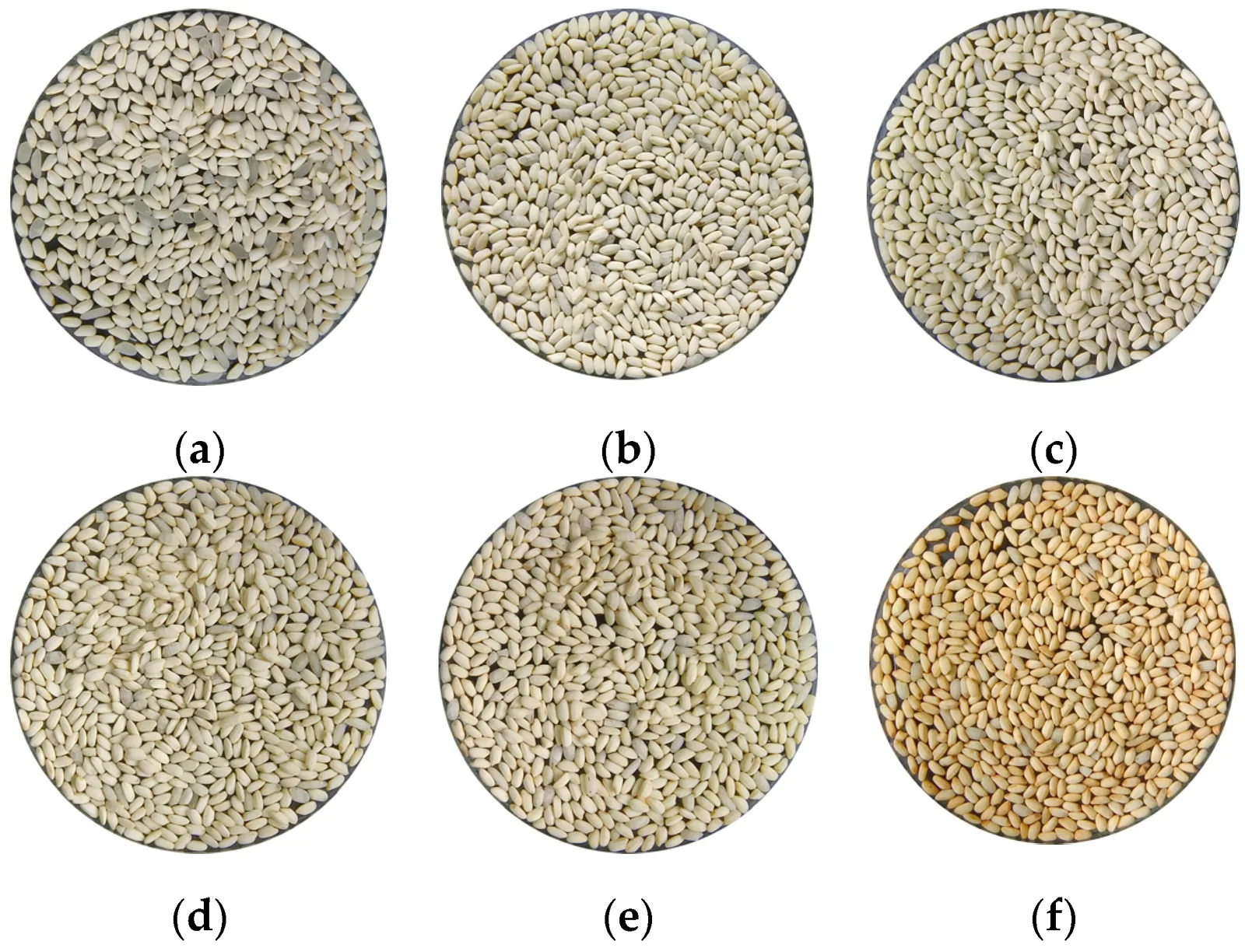

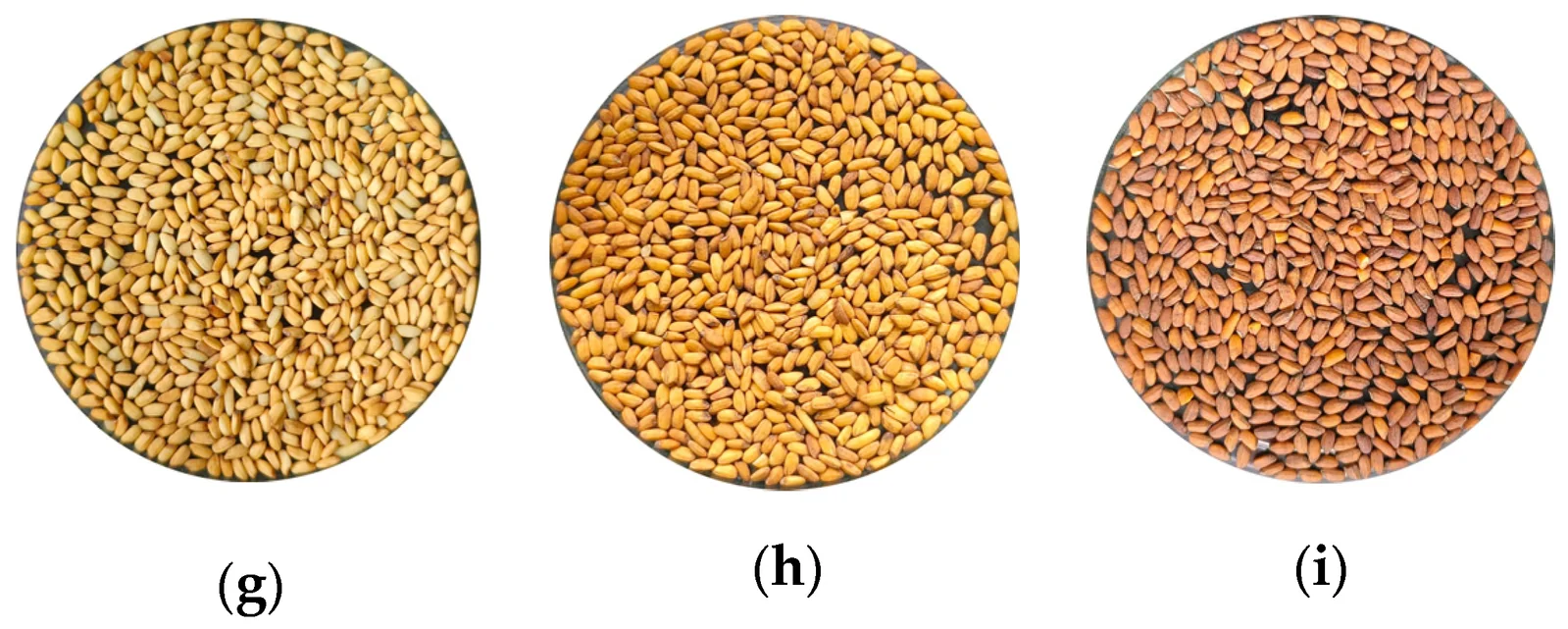
Macroscopic appearance of rice grains subjected to different roasting durations. (**a**) Control group (unroasted); (**b**–**i**) Samples roasted at 180 °C for 0, 10, 20, 30, 40, 50, 60, and 70 min, respectively, following a washing treatment. The images illustrate the progressive changes in surface color and texture induced by thermal processing.

The RS kinetic trend was further corroborated by the visual changes in the rice grains (Figure 2). In the initial stages (0–50 min), the grains turned from white to a uniform golden-yellow, indicating the progression of the Maillard reaction and starch gelatinization, which favors the formation of heat-stable structures such as amylose–lipid complexes or recrystallized starch. However, at 70 min, the grains exhibited a dark brown color, suggesting excessive thermal degradation or charring. This over-processing likely led to the hydrolysis of starch chains or the disruption of ordered crystalline structures, thereby reducing the measurable RS content.

### 2.3. Particle Size Distribution of Roasted Rice Flour

To further elucidate the role of particle size in modulating RS formation during roasting, the particle size distribution of roasted rice flour was analyzed. The particle size distribution of rice flour, as influenced by roasting temperature and sieve fractionation, is presented in Table 2. As expected, sieve size was the predominant factor determining particle size. For all temperature treatments (CK, R130, R150, and R180), the volume-weighted mean diameter (D [4,3]) and surface-weighted mean diameter (D [3,2]) progressively decreased as the sieve mesh size increased from 10 to 100. For instance, in the control group (CK), the D [4,3] value significantly dropped from 216.5 µm for the 10-mesh fraction to 55.95 µm for the 100-mesh fraction. Concurrently, the percentage of particles below 13.00 µm increased substantially with finer sieving, confirming the effectiveness of the fractionation process in obtaining distinct particle size ranges.

The roasting temperature also exerted a notable influence on the particle size of the rice flour. Compared to the control (CK), roasted samples generally exhibited smaller particle sizes within the same sieve fraction. This effect was particularly evident in the 10-mesh and 50-mesh fractions. For example, the D [4,3] of the R150-10 sample (146.5 µm) was significantly lower than that of the CK-10 sample (216.5 µm). This suggests that the thermal treatment may have altered the physical properties of the rice kernels, making them more brittle and susceptible to finer grinding.

Based on this analysis, the 100-mesh fractions were selected for all subsequent analyses of starch crystallinity, pasting properties (RVA), and digestibility. This selection was made to minimize the confounding physical effects of particle size, as the D [4,3] values for all 100-mesh samples (CK-100, R130-100, R150-100, and R180-100) were statistically similar (*p* > 0.05). By standardizing the particle size, any observed differences in starch properties can be more confidently attributed to the structural modifications induced by the roasting process itself, rather than variations in particle dimensions.

### 2.4. Starch Granule Morphology (SEM Analysis)

**Figure 3:**
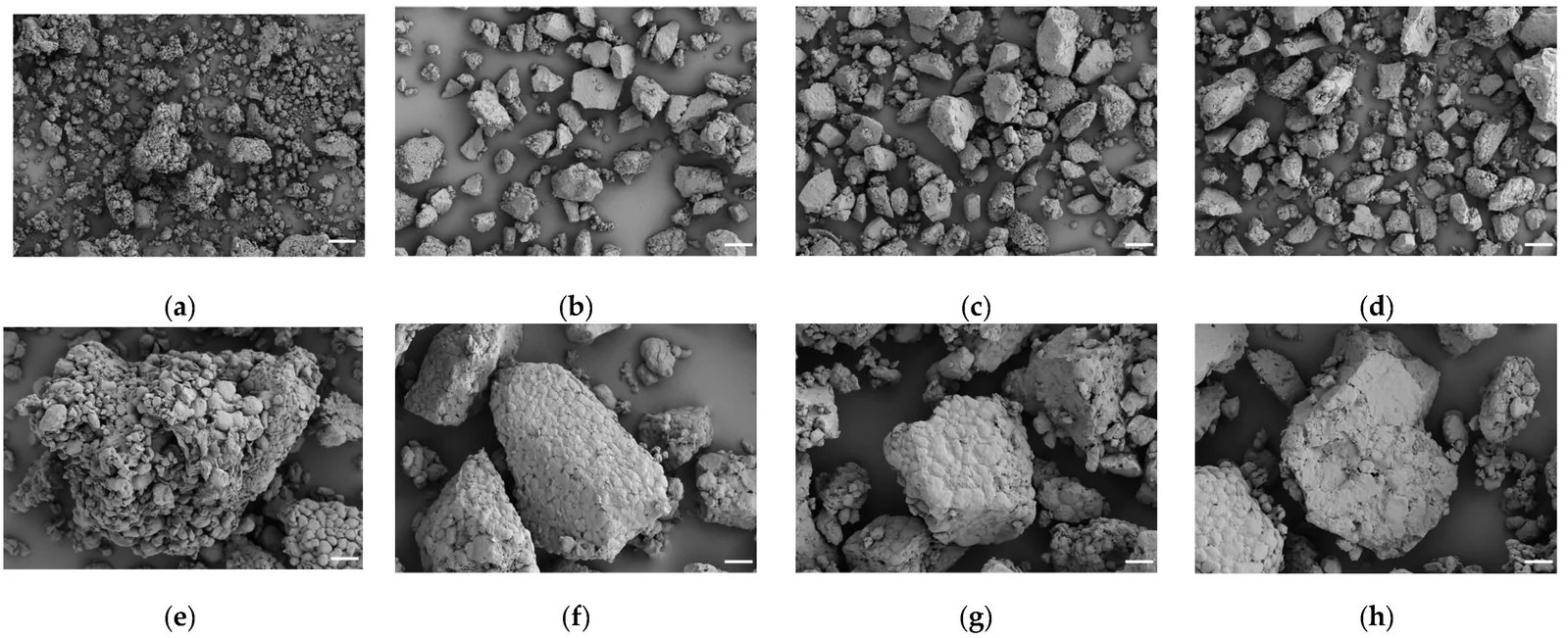
Scanning electron micrographs of high-resistant starches under various treatments: (**a**,**e**) untreated; (**b**,**f**) roasted at 130 °C; (**c**,**g**) roasted at 150 °C; (**d**,**h**) roasted at 180 °C. Magnifications are ×500 (**a**–**d**) and ×2000 (**e**–**h**); scale bars = 40 μm and 10 μm, respectively. All images were acquired with a Zeiss Sigma 360 field-emission SEM (Carl Zeiss AG, Oberkochen, Germany) at 3.00 kV accelerating voltage, 8.1–8.4 mm working distance, 0.1 pA probe current, and SE2 secondary-electron signal.

The microstructural changes in rice flour particles (100-mesh fraction) subjected to different roasting temperatures were observed using scanning electron microscopy (SEM), as shown in Figure 3. The untreated starch granules (Figure 3a,e) exhibited a typical polygonal shape with a relatively smooth and intact surface. The granules were discrete and loosely arranged, showing no signs of thermal damage or gelatinization. As the roasting temperature reached 130 °C (Figure 3b,f), the surface morphology began to change. The starch granules appeared slightly rougher compared to the control. Some small fragments adhered to the surface of larger granules, suggesting the initiation of structural loosening and partial disruption of the granule surface, possibly due to moisture loss and initial thermal stress. At 150 °C (Figure 3c,g), the morphological alterations became more pronounced. The distinct polygonal edges of the starch granules became blurred. The surface showed increased roughness and signs of aggregation, suggesting structural changes in the granule surface. In the sample roasted at 180 °C (Figure 3d,h), the original granular structure was largely replaced by fused, irregular lumps with a melted appearance. The starch granules appeared to have agglomerated into a continuous matrix with a porous texture. These morphological changes are consistent with thermal disruption of the granule surface, as further corroborated by the RVA and XRD results (see Section 2.5 and Section 2.6).

### 2.5. Pasting Properties (RVA) of Roasted Rice Flour

The pasting properties of rice flour, as influenced by roasting temperatures, were evaluated using the Rapid Visco Analyzer (RVA), with the results summarized in Table 3. The RVA profiles revealed a dramatic alteration in the thermal and rheological properties of the rice flour upon roasting. Compared to the control sample (CK-100), which exhibited a typical starch pasting curve with a high peak viscosity (1606 cP) and a distinct pasting temperature (87.8 °C), all roasted samples (R130-100, R150-100, and R180-100) showed negligible viscosity development. Specifically, the peak viscosity of the roasted samples decreased sharply to 244–264 cP, representing a reduction of over 85% compared to the control.

Furthermore, the pasting temperature was undetectable for the roasted samples, and the characteristic viscosity breakdown and setback observed in the control were absent. These results indicate that roasting significantly altered the pasting behavior of the starch. The reduced peak viscosity and absence of pasting temperature suggest that the starch granules had undergone structural modifications affecting their ability to swell and gelatinize during the RVA heating cycle. These observations are consistent with partial pre-gelatinization and molecular reorganization during roasting, as further supported by the XRD results (Section 2.6). Notably, at roasting temperatures ≥ 150 °C, the drastic collapse of pasting viscosity likely reflects the combined effects of heat-moisture treatment (HMT)-induced structural reorganization and thermal dextrinization, which fragments long starch chains into short glucans incapable of forming the extended entanglement networks required for viscosity development. The apparent paradox between increased relative crystallinity (XRD) and negligible shear resistance (RVA) is further discussed in Section 3.

### 2.6. Crystalline Structure Analysis (XRD)

**Figure 4:**
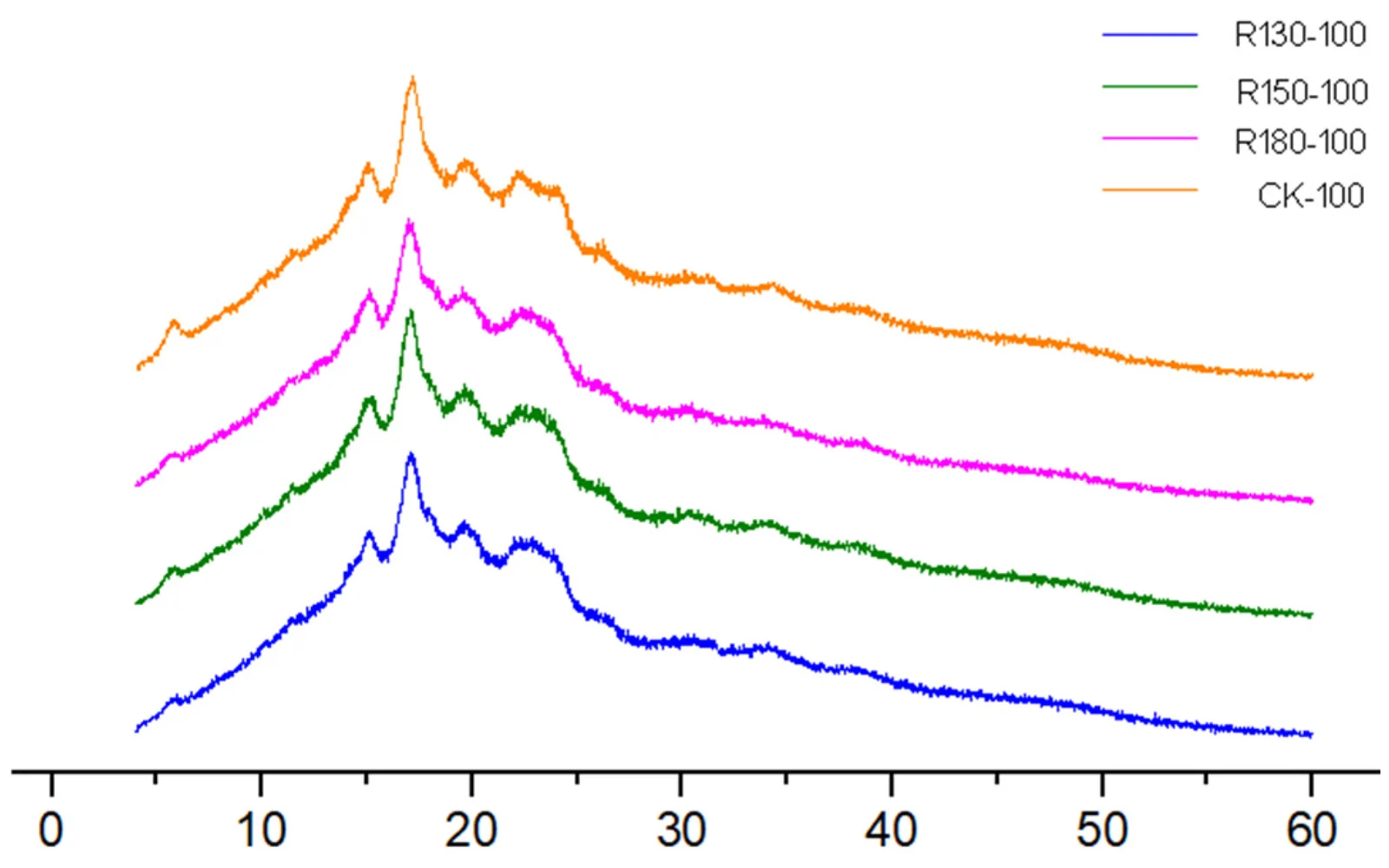
X-ray diffraction (XRD) patterns of different treatments.

The crystalline structure of rice flour subjected to different roasting temperatures was investigated using X-ray diffraction (XRD), and the results are presented in Figure 4 and Table 4. All samples, including the unroasted control and the roasted samples (R130-100, R150-100, and R180-100), exhibited a typical B-type diffraction pattern, characterized by strong peaks at around 17° and 23° (2θ) and a characteristic peak at 5.6°. This indicates that the fundamental crystalline structure of the rice starch was not altered by the roasting process, despite the thermal treatment.

However, the degree of crystallinity showed a significant increase with increasing roasting temperature. The unroasted control sample had a crystallinity of 19.07 ± 0.16%, which increased to 19.96 ± 0.28% for R130-100, 20.74 ± 0.46% for R150-100, and 19.71 ± 0.35% for R180-100. This trend suggests that the roasting process, particularly at higher temperatures, promoted the reorganization and ordering of the starch molecular chains within the amorphous regions, leading to an increase in the overall crystallinity.

The increase in crystallinity is likely attributed to the partial molecular gelatinization and subsequent retrogradation of starch during roasting. The thermal energy from roasting disrupts the amorphous regions of the starch granules, allowing the amylose and amylopectin chains to reassociate and form more ordered crystalline structures. This phenomenon is consistent with the observed changes in the RVA profiles, where the roasted samples exhibited significantly reduced viscosity and no detectable pasting temperature, indicating that the starch granules had undergone structural changes that affected their ability to swell and gelatinize.

### 2.7. Amylopectin Chain-Length Distribution

The chain length distribution analysis revealed that roasting significantly altered the starch fine structure compared to the unroasted control (*p* < 0.05), with distinct effects observed across different chain lengths (Table 5). Specifically, the proportion of short chains (DP 6–12) increased significantly after roasting, with the R150-100 sample exhibiting the highest value (14.68%) compared to the control (14.26%), indicating that thermal treatment promoted the formation of shorter chains; conversely, the proportion of very long chains (DP ≥ 37) decreased significantly, from 23.11% in the control to a minimum of 22.49% in the R150-100 sample, suggesting concurrent degradation of long chains. This trade-off was further supported by the significant reduction in average DP, which decreased from 26.11 in the unroasted sample to 25.84 in the R150-100 sample, confirming that roasting induced a net shortening of starch chains. Additionally, the proportion of intermediate chains (DP 13–24) increased with higher temperatures (peaking at 46.25% in R180-100), likely compensating for the loss of longer chains and contributing to the structural reorganization of the starch molecules.

The observed decrease in very long amylopectin chains (DP ≥ 37) concurrent with the increase in short chains (DP 6–12) provides important mechanistic insight into the RS increase during roasting. The thermal energy during roasting partially disrupts the amorphous regions of starch granules, mobilizing native amylose and long amylopectin chains to reassociate into more ordered double-helical structures. However, the concurrent decrease in DP ≥ 37 chains suggests that this process is counterbalanced by thermal cleavage, which generates short glucan fragments (DP 6–12) capable of reassociating into enzyme-resistant crystalline structures during cooling. This mechanism is consistent with the higher RS content observed at 150 °C, where the balance between chain cleavage and reorganization is optimal, whereas at 180 °C, excessive thermal degradation may produce fragments too short to form stable crystallites, explaining the decline in RS content.

Therefore, the RS increase in roasted Youtangdao 3 rice flour likely stems primarily from the structural reorganization of thermally induced short-chain glucans (DP 6–12) during cooling, rather than from native chain rearrangement alone. The decrease in DP ≥ 37 chains serves as an indirect marker of the thermal cleavage that generates the short-chain precursors contributing to enhanced enzyme resistance.

### 2.8. In Vitro Starch Digestibility

**Figure 5:**
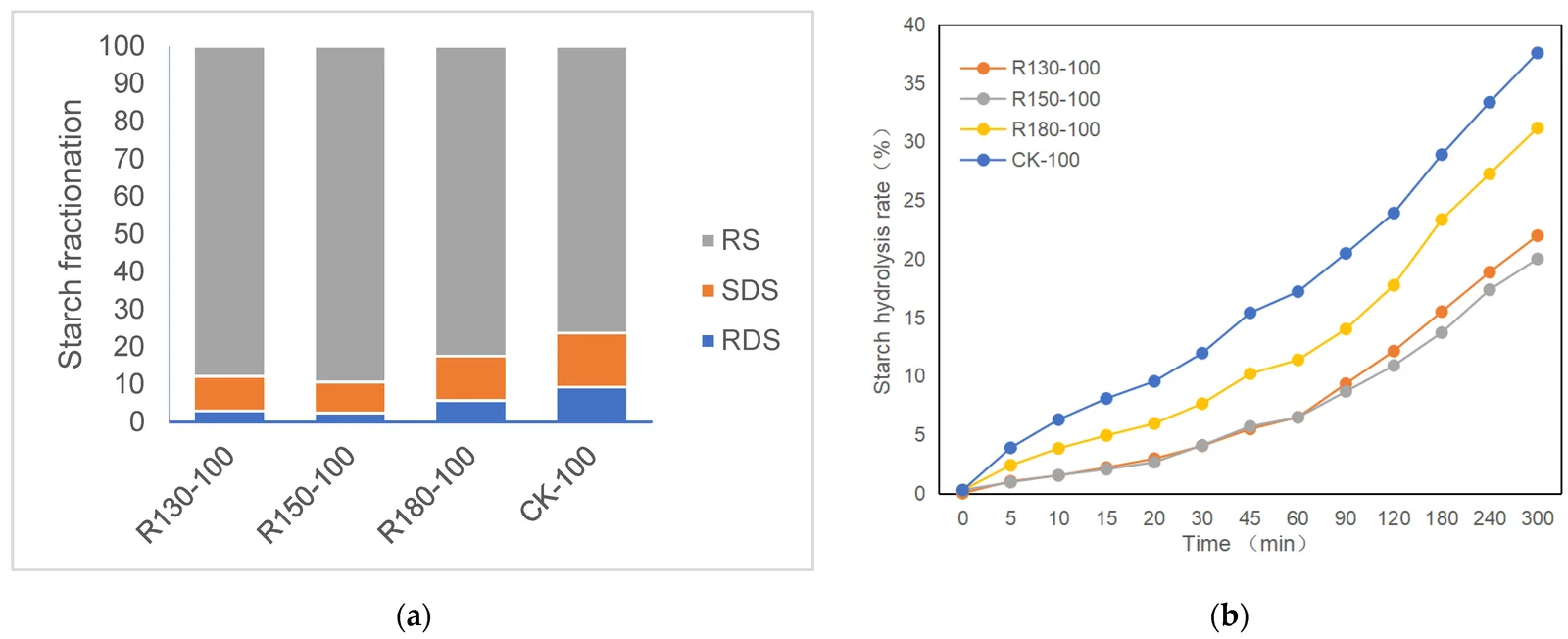
Effects of roasting temperature on the in vitro digestibility and hydrolysis kinetics of starch samples. (**a**) Starch fractionation showing the proportions of rapidly digestible starch (RDS), slowly digestible starch (SDS), and resistant starch (RS). (**b**) In vitro starch hydrolysis curves over time for samples roasted at different temperatures compared to the control group. CK-100: control group; R130-100, R150-100, R180-100: samples roasted at 130 °C, 150 °C, and 180 °C, respectively.

The in vitro digestibility profiles and hydrolysis kinetics of the starch samples subjected to different roasting temperatures (130 °C, 150 °C, and 180 °C) and the control group (CK-100) are presented in Table 6 and Figure 5. The results demonstrate that thermal treatment significantly altered the proportions of rapidly digestible starch (RDS), slowly digestible starch (SDS), and resistant starch (RS) compared to the control CK-100 (*p* < 0.05).

As shown in the stacked bar chart (Figure 5a) and detailed in Table 6, the CK-100 sample exhibited the highest content of RDS (9.30%) and SDS (14.37%), corresponding to the lowest RS content (76.33%). In contrast, all roasted samples showed a marked reduction in digestible fractions and a substantial increase in RS content. Specifically, the RDS content decreased to 2.95%, 2.39%, and 5.70% for R130-100, R150-100, and R180-100, respectively. Among the roasted samples, R150-100 achieved the most significant transformation, exhibiting the lowest RDS (2.39%) and SDS (8.26%) contents, while reaching the peak RS level of 89.36%. However, when the temperature increased to 180 °C (R180-100), the RS content declined to 82.50%, indicating a non-linear relationship between roasting temperature and retrogradation efficiency.

These compositional changes were further corroborated by the starch hydrolysis rates (Figure 5b). The hydrolysis rate of the CK-100 sample was the fastest, reaching approximately 37.61% digestibility within 300 min. Conversely, the roasted samples displayed significantly retarded digestion rates. Notably, the R150-100 sample showed the slowest hydrolysis curve, maintaining the lowest cumulative digestion percentage throughout the entire incubation period, which aligns with its highest RS content observed in Table 6.

## 3. Discussion

### 3.1. Thermal Treatment Effects on RS Formation and Structural Reorganization

As the core step of the modification, the pre-roasting moisture adjustment (approximately 24% moisture content) combined with thermal treatment at 150 °C established a critical moist heat reaction environment. Unlike dry heating, the water retained within the rice grains acted as a plasticizer during heating, promoting moderate swelling of the starch granules without completely disrupting their crystalline lattices [16]. Starch retrogradation is fundamentally defined as a process in which disaggregated amylose and amylopectin chains within a gelatinized starch paste reassociate to form more ordered structures [21]. We hypothesize that the controlled moist heat environment in this study significantly lowered the energy barrier for molecular chain mobility, thereby inducing the reorganization of amylose from disordered amorphous regions into ordered structures and inducing the formation of RS. The increase in crystallinity observed in the X-ray diffraction (XRD) analysis corroborates this hypothesis, indicating that the roasting process is not merely physical drying, but a chemical modification process that induces phase transitions and structural reorganization in starch, thereby laying the material foundation for the formation of resistant starch.

The apparent contradiction between the SEM/RVA and XRD results can be reconciled by considering the differential effects of roasting on the amorphous and crystalline regions of starch granules. Roasting at moderate temperatures (130–150 °C) with limited moisture primarily affects the amorphous regions, where amylose and the amorphous lamellae of amylopectin are more susceptible to thermal disruption. The SEM observations of surface roughening and the RVA results indicating loss of gelatinization ability reflect disruption of the amorphous outer layer. Concurrently, the XRD results showing preservation of the B-type crystalline pattern and increased relative crystallinity are consistent with the selective disruption of amorphous regions, which increases the proportion of crystalline material. This phenomenon has been reported in heat-moisture treated starches, where relative crystallinity increases despite partial granular disruption. Therefore, the simultaneous occurrence of morphological disruption, reduced pasting ability, crystalline polymorphism preservation, and increased relative crystallinity is not contradictory but rather reflects the heterogeneous nature of roasting effects: surface/amorphous disruption coexists with crystalline region stabilization and reorganization. This interpretation is consistent with previous reports on heat-moisture treated rice starch [13] and high-amylose maize starch [22].

Integrating the results from XRD, SEM, RVA, amylopectin chain-length distribution, and in vitro digestibility analyses, the observed increase in RS content upon roasting can be attributed to multiple complementary mechanisms. First, the increased relative crystallinity (XRD, Table 4) and the increased proportion of short amylopectin chains (DP 6–12, Table 5) suggest that roasting promoted molecular reassociation and reorganization of starch chains into more enzyme-resistant crystalline structures. Short amylopectin chains (DP 6–12) are known to form tight double helices that resist enzymatic hydrolysis. Second, the RVA results (reduced pasting, absent breakdown) indicate that partial pre-gelatinization and subsequent retrogradation during roasting created a more ordered structure with reduced swelling capacity, limiting enzymatic accessibility. Third, the SEM observations of surface roughening and morphological changes suggest the formation of physical barriers that may hinder enzyme diffusion into the granule interior. Fourth, the formation of amylose–lipid complexes during thermal treatment, as reported by Ma et al. (2026) [17], may contribute to RS formation, although this was not directly measured in the present study. Overall, the increase in RS is likely the result of the combined effects of molecular chain reassociation, increased relative crystallinity, reduced enzymatic accessibility due to structural reorganization, and potentially amylose–lipid complex formation, rather than a single mechanism.

Importantly, the beneficial effects of roasting on RS formation were not monotonic with temperature. However, when the temperature was further elevated to 180 °C, the RS content showed a declining trend. This may be attributed to thermal degradation of long starch chains caused by excessive thermal processing, which may have disrupted the molecular chain length required to form long-range ordered crystals, thereby weakening the enzyme resistance. This indicates that moderate thermal processing is crucial for optimizing digestive properties, as excessively high temperatures may instead compromise the structural integrity of functional components [22].

### 3.2. Role of Particle Size in RS Retention and Digestibility

In addition to thermal treatment, the particle size grading after milling exerted a notable impact on the in vitro digestion results [23]. Under identical thermal treatment conditions, coarser particles (10-mesh) retained a higher RS content and exhibited a lower enzymatic hydrolysis rate compared to finer particles (100-mesh). This finding reveals the critical role of the “specific surface area effect” in the digestion process: although the fine particles originated from the modified rice grains, their large specific surface area significantly increased the probability of enzyme–substrate contact, thereby accelerating the enzymatic reaction. This observation aligns with previous studies, which demonstrated that reducing particle size enhances the accessibility of digestive enzymes to the starch matrix, consequently leading to higher hydrolysis rates [24]. Conversely, the larger particle size of the coarse fraction restricted the diffusion and penetration of digestive enzymes into the interior of the particles. This physical diffusion limitation was manifested in a reduced digestion rate and an increased measured RS content. Therefore, the coarse particle size further protected the resistant structure induced by thermal treatment at the physical level, delaying the hydrolysis process.

### 3.3. Digestibility Characteristics and Methodological Considerations

In terms of digestive properties, thermal treatment significantly altered the enzymatic hydrolysis kinetics of the rice flour. As the roasting temperature increased, the content of rapidly digestible starch (RDS) decreased substantially, while the contents of slowly digestible starch (SDS) and resistant starch (RS) increased significantly. Particularly under the 150 °C condition, the samples exhibited optimal anti-digestive performance. It should be noted that two different analytical methods were employed for RS determination in this study, each serving a distinct purpose. The AOAC 2002.02 method (Megazyme© RS Assay Kit, Bray, County Wicklow, Ireland) was used for the quantitative screening of RS content across all temperature–mesh combinations (Table 1), providing a standardized measure of total RS after 16 h enzymatic hydrolysis. The modified Englyst method was used for the detailed fractionation of starch digestibility (RDS, SDS, and RS) in the 100-mesh samples (Table 6), providing information on the kinetic aspects of starch hydrolysis with RS defined as the fraction remaining after 120 min of hydrolysis. The RS values obtained by the two methods are not directly comparable due to differences in enzyme systems and hydrolysis durations. All conclusions regarding RS content across different temperature–mesh combinations are based on the AOAC method (Table 1), which provides the absolute RS quantification, while conclusions regarding digestibility fractions and hydrolysis kinetics are based on the Englyst method (Table 6).

### 3.4. Limitations and Future Directions

Several limitations of this study should be acknowledged. First, only in vitro starch digestibility was evaluated; no predicted glycemic index (pGI), in vivo glycemic response, or sensory evaluation were performed. Therefore, the results provide a preliminary basis for low-GI food applications but do not constitute direct evidence of glycemic benefits in vivo. Second, the structural and digestibility analyses were conducted exclusively on 100-mesh samples; the interaction between roasting conditions and particle size at the structural level was not fully investigated. Third, the proposed mechanisms for RS formation are based on indirect evidence, and complementary analyses (differential scanning calorimetry, DSC; Fourier-transform infrared spectroscopy, FTIR) were not performed. Fourth, storage stability and processing scalability were not assessed. Future studies should include (i) in vivo glycemic response testing and pGI determination, (ii) structural analyses across multiple particle-size fractions, (iii) DSC and FTIR characterization to validate the proposed mechanisms, (iv) sensory evaluation and storage stability testing, and (v) pilot-scale processing trials to assess industrial feasibility.

## 4. Materials and Methods

### 4.1. Materials

The high-RS rice variety used in this study, known as Youtangdao 3, provided by the Shanghai Academy of Agricultural Sciences in China, has an RS content of up to approximately 13%. This variety was cultivated in the field during the 2025 growing season in Shanghai, China.

### 4.2. Sample Preparation

**Figure 6:**
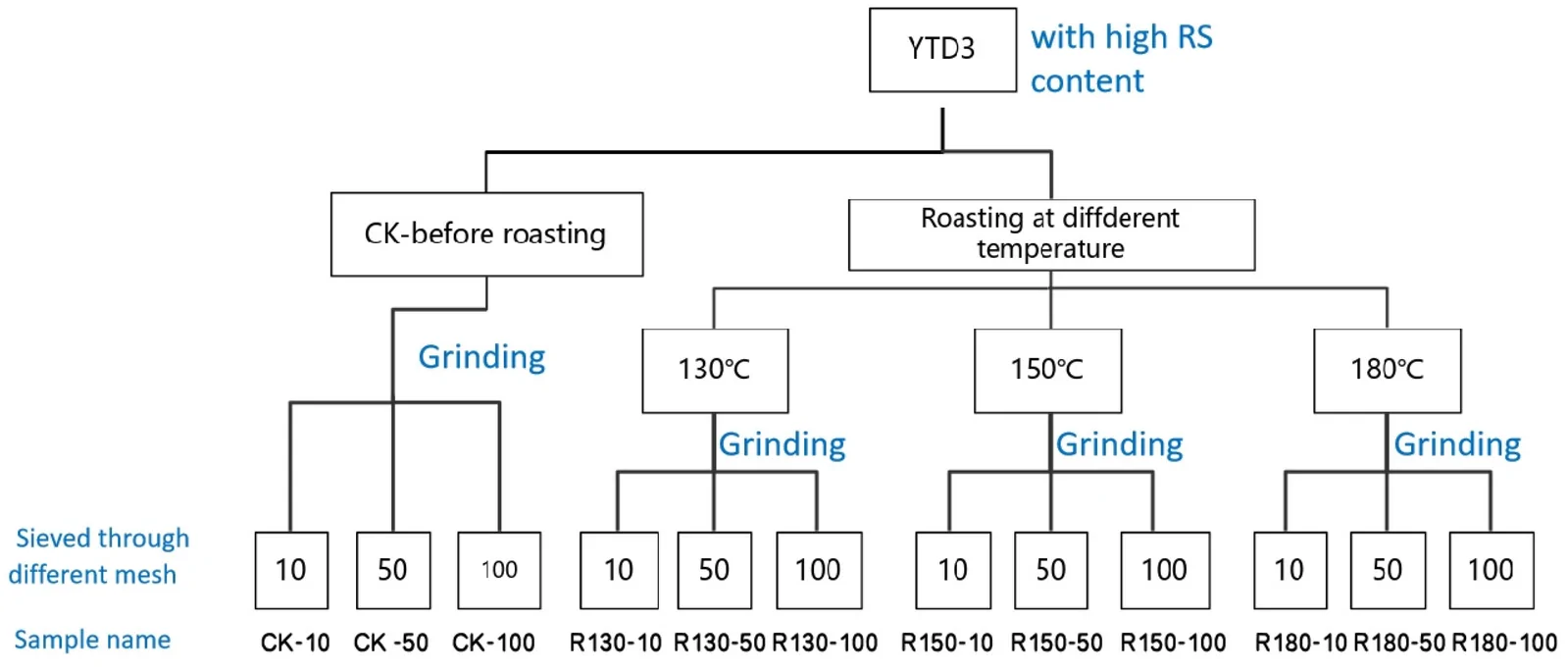
Sample preparation. YTD3, Youtangdao 3 rice variety with high RS content; CK, untreated control.

The preparation process of the roasted rice flour samples is illustrated in Figure 6. Briefly, whole rice grains were hydrated by mixing with distilled water at a ratio of 85:15 (*w*/*w*, 200 g rice to 30 g water). The rice grains had an inherent moisture content of 13.2 ± 0.5%. The mixture was equilibrated at room temperature (RT) for at least 1 h under a relative humidity of 40–60% to ensure uniform water absorption and grain softening. The total moisture content of the hydrated grains was approximately 24%, which falls within the typical moisture range for heat-moisture treatment of cereal grains. The hydrated rice grains were then divided into four groups for thermal treatment. One group served as the control (CK) and was processed without high-temperature roasting (or subjected to mild drying), while the other three groups were roasted at different temperatures: 130 °C, 150 °C, and 180 °C, respectively [16]. All roasting treatments were performed using an industrial roasting machine (MSDC-5, MS, Changzhou, China). For each batch, 200 g of hydrated rice grains (after water equilibration) were loaded into the roasting chamber. The machine was preheated to the target temperature for at least 30 min prior to sample loading. Temperature fluctuations during the process were maintained within ±3 °C of the set point. Preliminary optimization experiments were conducted to determine the optimal roasting conditions at each temperature. Rice grains were sampled at regular intervals during roasting, and the RS content, color parameters (CIE L*a*b*), and moisture content were monitored. The optimal roasting time was defined as the point at which the RS content reached its maximum while the grains maintained a uniform golden-yellow appearance without visible charring. Specifically, the b* value (yellowness) was used as an objective color indicator, increasing progressively from 21.10 ± 0.40 at 10 min to 34.08 ± 0.51 at 40 min. The endpoint was confirmed when the moisture content of the grains stabilized below 5%, corresponding to the color parameters of L = 71.76 ± 0.54, a = 7.86 ± 0.34, and b* = 34.08 ± 0.51.

Following the thermal treatment, all samples (including the control) were ground and passed through stainless steel sieves with aperture sizes of 10-mesh, 50-mesh, and 100-mesh to obtain fractions with distinct particle size distributions. The final samples were coded based on their treatment temperature and sieve size. For instance, samples roasted at 130 °C, 150 °C, and 180 °C and sieved through 10, 50, or 100 meshes were labeled as R130-10, R130-50, R130-100; R150-10, R150-50, R150-100; and R180-10, R180-50, R180-100 respectively (Figure 6). The unroasted control samples sieved through 10, 50, and 100 meshes were designated as CK-10, CK-50, and CK-100.

To investigate the kinetic changes in RS content during high-temperature roasting, an additional time-course experiment was conducted at 180 °C. Hydrated rice grains (200 g per batch, prepared as described above) were roasted in an oven at 180 °C. Samples were withdrawn at 0, 10, 20, 30, 40, 50, 60, and 70 min. Upon withdrawal, each sample was immediately cooled to room temperature on aluminum foil to halt further thermal reactions. The cooled samples were then ground and passed through a 100-mesh sieve. The RS content of each time-point sample was determined using the Megazyme RS assay kit (AOAC 2002.02) as described in Section 4.5. Three independent biological replicates were performed for each time point (*n* = 3).

### 4.3. Particle Size Measurement

The particle size distribution was determined using a laser diffraction particle size analyzer (Mastersizer 3000, Malvern Instruments Ltd., Worcestershire, UK). Approximately 0.5–1.0 g of rice flour was dispersed in 500 mL of distilled water (the dispersing medium) in the instrument’s wet dispersion unit. The sample was added gradually until the obscuration rate reached 5–8%. The dispersion was stirred at 2500 rpm throughout the measurement to maintain uniform suspension and prevent particle aggregation. No ultrasound was applied to avoid fragmentation of starch granules. The measurement duration was 12 s per reading, and five readings were averaged for each sample. Three independent measurements were performed for each treatment (*n* = 3). The optical model used was the Mie scattering theory, with the refractive index of starch particles and the dispersant (distilled water) set at 1.50 and 1.33, respectively, and the absorption index set at 0.1. The data were evaluated using Mastersizer 3000 software (Malvern Instruments Ltd., Worcestershire, UK, version 3.62). Water was selected as the dispersing medium because it is the most relevant medium for food applications and is consistent with the wet-milling context of rice flour processing. The main measurement indicators included the volume-weighted mean diameter (D [4,3]) and the surface area-weighted mean diameter (D [3,2]), as well as the percentage of particles below different particle sizes (76.00 μm, 45.00 μm, and 13.00 μm). The results for each sample were presented as the mean ± standard deviation to reflect the reliability and variability of the data (*n* = 3).

### 4.4. SEM (Scanning Electron Microscope) Analysis

The microstructure of the starch samples was observed using a scanning electron microscope (S-3400N, Hitachi, Tokyo, Japan). Prior to observation, samples were prepared to remove surface debris and ensure conductivity. Briefly, 100 mg of sample was suspended in 1 mL of distilled water in a 2 mL centrifuge tube and vortexed until uniformly mixed. The suspension was centrifuged at 5000× *g* for 1 min, and the supernatant was discarded. This washing process was repeated five times to remove soluble sugars and small particles that could obscure the granule surface. Subsequently, 1 mL of anhydrous ethanol was added to the tube and vortexed to facilitate dehydration and reduce surface tension effects during drying. A small amount of the prepared suspension was transferred onto a copper stage using a 200 μL pipette and spread as evenly as possible. The samples were dried overnight at 37 °C to minimize thermal damage to the starch structure.

To enhance conductivity, the dried samples were sputter-coated with a thin layer of gold (~10 nm) using an ion sputter coater (E-1010, Hitachi). Observations were conducted at an accelerating voltage of 15 kV and a working distance of 10–15 mm. For each treatment, at least five independent fields of view were examined at magnifications of 500×, 1000×, and 2000×. Representative images from at least three independently prepared samples are presented.

### 4.5. RS Content Analysis

The RS content was measured according to the AOAC Method 2002.02 and AACC Method 32-40.01, using an RS analysis kit (Megazyme, Ltd., Bray, Wicklow, Ireland). Briefly, samples were incubated with pancreatic α-amylase and amyloglucosidase at 37 °C for 16 h. The undigested starch was precipitated with ethanol, dissolved in 2 M KOH, and hydrolyzed with amyloglucosidase. The released glucose was measured using a glucose oxidase–peroxidase reagent. This method measures RS as the starch fraction resistant to enzymatic hydrolysis after 16 h of incubation and was used for the quantitative screening of RS content across all temperature–mesh combinations (Table 1). Three independent biological replicates were performed (*n* = 3).

### 4.6. XRD

X-ray diffraction (XRD) patterns were collected on a D/max 2200PC X-ray diffractometer (Rigaku Corporation, Tokyo, Japan). The X-ray intensity was measured using a NaI crystal scintillation counter with a scanning range of 4° to 60°, a step size of 0.02°, and a scanning speed of 4°/min. Based on the intensity of diffraction peaks at different diffraction angles, the MDI Jade 6 software was used to calculate the starch crystallinity.

### 4.7. Starch Pasting Properties

Starch pasting properties were determined using the Rapid Visco Analyzer (RVA-Tec Master, Perten, Sweden). This analysis measures changes in the viscosity of starch slurry during heating and cooling. Approximately 3 g of each starch sample (corrected to 14% wet basis) was mixed with 25 mL of water and then added to the RVA canister for testing. A programmed heating and cooling cycle was used at a constant shear rate. The temperature of the starch paste reached 50 °C in 1 min, was heated to 95 °C in 4 min and 45 s, maintained at 95 °C for 2 min and 30 s, then cooled to 50 °C in 11 min, and maintained for 2 min; at 13 min, the process was terminated. After that, the starch was gelatinous. Time to peak viscosity (Ptime), pasting temperature (PT), peak viscosity (PV), trough viscosity (TV), breakdown viscosity (BV), final viscosity (FV), setback viscosity (SV) were automatically analyzed by the analyzer’s software. Three independent replicates were performed for each group (*n* = 3).

### 4.8. Amylopectin Chain-Length Distribution Analysis

Starch extraction: Rice flour (100-mesh, 2 g) was defatted with 80% (*v*/*v*) methanol at 40 °C for 2 h, and the residue was dried at 40 °C overnight. The defatted flour was suspended in 40 mL of 0.05 M NaOH containing 0.02% (*w*/*v*) sodium azide and stirred at room temperature for 4 h. The suspension was centrifuged at 4000× *g* for 15 min, and the supernatant was adjusted to pH 7.0 with 0.5 M HCl. The starch was precipitated by adding 3 volumes of absolute ethanol, centrifuged at 4000× *g* for 15 min, washed twice with 80% ethanol, and dried at 40 °C.

Extracted starch (10 mg, dry basis) was dissolved in 5 mL water in a boiling water bath for 60 min with continuous stirring. Then, 50 μL of sodium acetate (0.6 M, pH 4.4), 10 μL of NaN_3_ (2% *w*/*v*), and 10 μL of isoamylase (1400 U) were added, and the mixture was incubated at 37 °C for 24 h. A 0.5% (*w*/*v*) sodium borohydride solution was added, mixed by vortexing, and allowed to stand for 20 h. Then, 600 μL was transferred to a centrifuge tube and dried under nitrogen at room temperature. The dried sample was dissolved in 30 μL of 1 M NaOH for 60 min, diluted with 570 μL water, centrifuged at 12,000 rpm for 5 min, and the supernatant was transferred for analysis. The chromatographic system employed a Thermo ICS5000+ ion chromatography system (ICS5000+, Thermo Fisher Scientific, Waltham, MA, USA), utilizing an electrochemical detector for the analysis of starch. A Dionex™ CarboPac™ PA200 column (250 × 4.0 mm, 10 μm, Dionex, Chelmsford, MA, USA) was used, with an injection volume of 5 μL. The mobile phase consisted of Phase A: 0.2 M NaOH; Phase B: 0.2 M NaOH containing 0.2 M NaAc. The column temperature was maintained at 30 °C, and the components were analyzed using the electrochemical detector. The flow rate was set at 0.4 mL/min. The elution gradient was as follows: 0 min—A/B (90:10, *V*/*V*); 10 min—A/B (90:10, *V*/*V*); 30 min—A/B (40:60, *V*/*V*); 50 min—A/B (40:60, *V*/*V*); 50.1 min—A/B (90:10, *V*/*V*); and 60 min—A/B (90:10, *V*/*V*).

The system was calibrated using a mixture of maltooligosaccharide standards (DP 1–12, Sigma-Aldrich, St. Louis, Mo, USA). Chain-length distribution was expressed as the molar percentage of each degree of polymerization (DP) range: DP 6–12, DP 13–24, DP 25–36, and DP ≥ 37. Three independent replicates were performed (*n* = 3).

### 4.9. Determination of Starch Digestibility

The in vitro starch digestibility was determined according to the method described by Englyst et al. with slight modifications [25]. Briefly, 200 mg of the sample was weighed into a 50 mL centrifuge tube containing 5 glass beads and 20 mL of 0.2 mol/L sodium acetate buffer (pH 5.2). After cooling to room temperature, 10 mL of enzyme solution containing porcine pancreatic α-amylase (290 U/mL) and amyloglucosidase (15 U/mL) was added. The mixture was incubated in a water bath shaker at 37 °C with a shaking speed of 150 r/min. Aliquots of 1 mL were withdrawn at specific time intervals (0, 20, 40, 60, 80, 100, 120, 180, 240, and 300 min). The enzymatic reaction was immediately stopped by placing the aliquots in a boiling water bath for 5 min, followed by centrifugation at 4000 r/min for 5 min. The supernatant was then used for glucose content determination using the D-Glucose Assay Kit. Finally, the RDS, SDS, and RS contents of the endosperm were determined using the following formulas:

$$Starch\;hydrolysis\;rate\;(\%)=\frac{DS\times 0.9}{TS}\times 100\%$$

where DS represents the glucose content (mg) and TS represents the total starch content (mg) of the sample.

The relative content of rapidly digestible starch (RDS), slowly digestible starch (SDS), and resistant starch (RS) was calculated based on the glucose released after 20 min (G_20_) and 120 min (G_120_) of hydrolysis, with G_0_ being the glucose content at time zero, as shown in the following equations:

$$RDS\;(\%)=\frac{(G_{20}{-G}_{0})\times 0.9}{TS}\times 100\%$$

$$SDS\;(\%)=\frac{(G_{120}{-G}_{20})\times 0.9}{TS}\times 100\%$$

$$\begin{array}{c} RS\;(\%)=\frac{\left(TS-RDS-SDS\right)}{TS}\times 100\% \end{array}$$

Note: The RS values obtained by the AOAC 2002.02 method (Table 1) and the modified Englyst method (Table 6) are not directly comparable due to differences in enzyme type and concentration, hydrolysis duration (16 h vs. 120 min), and calculation principles. The AOAC method was used for quantitative RS screening across all temperature–mesh combinations, while the Englyst method was used for detailed digestibility fractionation of the 100-mesh samples.

### 4.10. Statistical Analysis

All experiments were performed with at least three independent biological replicates (*n* = 3), each representing a separate batch of sample preparation. Technical replicates (instrumental repeated measurements) were averaged before statistical analysis. The experimental unit was defined as one independently prepared sample batch. For the RS content data (Table 1), which involves two experimental factors (roasting temperature and particle size), data were analyzed by two-way ANOVA to evaluate the main effects of roasting temperature, particle size, and their interaction. For the structural and digestibility analyses conducted on 100-mesh samples only (Table 3, Table 4, Table 5 and Table 6, Figure 3, Figure 4 and Figure 5), data were analyzed by one-way ANOVA with roasting temperature as the single factor. Post hoc multiple comparisons were performed using Tukey’s HSD test. All statistical analyses were performed using SPSS software (version 26.0, IBM Corp., Armonk, NY, USA). Differences were considered statistically significant at *p* < 0.05.

## 5. Conclusions

In conclusion, this study demonstrates that the synergistic application of moist roasting and particle size control significantly enhances the resistant starch (RS) content and modifies the digestibility of Youtangdao 3 rice flour. Crucially, the results reveal divergent optimal temperatures depending on whether the priority is maximizing absolute RS yield or optimizing starch conversion efficiency. Specifically, roasting at 130 °C proved optimal for maximizing the absolute accumulation of RS, achieving the highest content (27.45% via AOAC method). This suggests that moderate thermal treatment favors the preservation of starch substrate while promoting sufficient structural rearrangement for RS formation. Conversely, treatment at 150 °C yielded the highest proportion of RS relative to total starch (89.36%) and the lowest rapidly digestible starch (RDS) fraction. Although the absolute RS content at 150 °C was slightly lower due to potential thermal degradation of the starch matrix, this temperature facilitated the most efficient conversion of available starch into resistant forms, resulting in superior resistance to enzymatic hydrolysis. Therefore, the selection of processing conditions should be application-driven: 130 °C is recommended for producing high-RS functional ingredients where total yield is paramount, whereas 150 °C is preferable for applications requiring maximal suppression of glycemic response and high structural purity. These findings provide a theoretical basis for the precise thermal processing of rice flour, though future in vivo studies are warranted to validate the physiological efficacy of these distinct RS profiles.

## Figures and Tables

**Table 1:** Effects of roasting temperature and particle size on resistant starch (RS) content and two-way ANOVA results. (**A**) RS content of each treatment group; (**B**) two-way ANOVA results.

(**A**)					
**Treatments**	**Roasting Temp. (°C)**	**Particle Size (Mesh)**		**RS content (%)**	**Roasting Time**
R130-10	130	10		27.45 ± 0.24 a	100 min
R130-50	130	50		26.35 ± 0.73 ab	100 min
R130-100	130	100		25.79 ± 0.28 bc	100 min
R150-10	150	10		25.30 ± 0.77 cd	70 min
R150-50	150	50		23.64 ± 1.02 de	70 min
R150-100	150	100		22.75 ± 0.95 ef	70 min
R180-10	180	10		24.24 ± 0.62 cde	50 min
R180-50	180	50		23.28 ± 0.52 def	50 min
R180-100	180	100		21.98 ± 0.41 f	50 min
CK-10	/	10		13.87 ± 0.73 g	/
CK-50	/	50		11.39 ± 0.73 h	/
CK-100	/	100		11.40 ± 0.16 h	/
(**B**)					
**Source of Variation**	**SS**	**df**	**MS**	**F**	***p***
Roasting Temperature	840.39	2	420.20	30.06	<0.001
Particle Size	423.69	2	211.85	15.15	<0.001
Temperature × Size	50.44	4	12.61	0.90	0.483
Error	251.79	18	13.99		
Total	1566.31	26			

Note: Values are expressed as mean ± SD (*n* = 3). Different lowercase letters indicate significant differences among treatments (*p* < 0.05). Two-way ANOVA was performed to evaluate the main effects of roasting temperature and particle size, as well as their interaction, on RS content. indicates *p* < 0.001.

**Table 2:** Particle size distribution of rice flour samples subjected to different roasting temperatures and sieve fractionations.

Treatment	D [4,3] (μm)	D [3,2] (μm)	Percentage Below 76.00 μm (%)	Percentage Below 45.00 μm (%)	Percentage Below 13.00 μm (%)
R130-10	152.5 ± 2.12 a	33.55 ± 0.21 b	51.16 ± 0.37 c	35.41 ± 0.27 c	11.94 ± 0.11 b
R130-50	128 ± 0.00 b	37.9 ± 0.28 a	54.35 ± 0.15 b	37.89 ± 0.07 b	9.72 ± 0.19 c
R130-100	53.6 ± 0.56 c	24.00 ± 0.14 c	80.97 ± 0.49 a	58.41 ± 0.41 a	16.34 ± 0.12 a
R150-10	146.5 ± 2.12 a	36.65 ± 0.21 a	51.68 ± 0.01 c	35.45 ± 0.08 c	10.22 ± 0.12 b
R150-50	121 ± 0.00 b	36.85 ± 0.07 a	55.76 ± 0.12 b	38.09 ± 0.02 b	9.68 ± 0.08 c
R150-100	53.75 ± 0.07 c	25.15 ± 0.07 b	81.11 ± 0.11 a	57.38 ± 0.06 a	15.15 ± 0.09 a
R180-10	168.5 ± 2.12 a	39.7 ± 0.28 a	48.31 ± 0.23 c	32.13 ± 0.12 c	9.11 ± 0.14 b
R180-50	137.5 ± 0.70 b	38.95 ± 1.06 a	50.58 ± 0.15 b	34.78 ± 0.28 b	9.51 ± 0.58 b
R180-100	53.9 ± 0.28 c	23.95 ± 0.35 b	80.76 ± 0.01 a	56.76 ± 0.01 a	16.67 ± 0.56 a
CK-10	216.5 ± 0.71 a	32.7 ± 1.13 a	46.83 ± 0.08 c	36.49 ± 0.21 c	14.18 ± 0.66 c
CK-50	122 ± 0.00 b	26.45 ± 0.35 b	56.43 ± 0.19 b	43.96 ± 0.26 b	18.09 ± 0.40 b
CK-100	55.95 ± 0.21 c	21.8 ± 0.14 c	76.63 ± 0.08 a	54.30 ± 0.01 a	21.58 ± 0.17 a

Note: D [4,3] is the volume weight mean diameter, D [3,2] is the surface area weighted mean diameter; Values are expressed as mean ± SD (*n* = 3). Values with different lowercase letters within the same column indicate significant differences among different roasting temperatures within each sieve fraction (*p* < 0.05).

**Table 3:** Pasting viscosity of high-resistant rice under various treatments.

	PV (cP)	TV (cP)	BV (cP)	FV (cP)	SV (cP)	PT (°C)
R130-100	264 ± 8 b	270 ± 9 b	−6 ± 2 b	528 ± 14 b	264 ± 7 b	/
R150-100	244 ± 5 c	249 ± 4 c	−5 ± 2 b	492 ± 11 c	248 ± 5 b	/
R180-100	254 ± 6 b	260 ± 7 b	−6 ± 2 b	501 ± 12 b	247 ± 9 b	/
CK-100	1606 ± 50 a	1492 ± 45 a	114 ± 8 a	2579 ± 80 a	973 ± 36 a	87.8 ± 2.5

Note: The results were expressed as mean ± SD (*n* = 3). The different lowercase letters indicate significant differences among treatments (*p* < 0.05). PV, peak viscosity; TV, trough viscosity; BV, breakdown viscosity; FV, final viscosity; SV, setback viscosity; PT, pasting temperature.

**Table 4:** Crystallinity and crystal patterns of the samples.

Sample	Degree of Crystallinity (%)	Crystal Pattern
CK-100	19.07 ± 0.16 c	B
R130-100	19.96 ± 0.28 b	B
R150-100	20.74 ± 0.46 a	B
R180-100	19.71 ± 0.35 bc	B

Note: Values with different lowercase letters in the same column indicate significant differences (*p* < 0.05).

**Table 5:** Chain length distribution of amylopectin of debranched starches.

Sample	DP 6–12 (%)	DP 13–24 (%)	DP 25–36 (%)	DP ≥ 37 (%)	Average DP
R130-100	14.38 ± 0.01 b	46.15 ± 0.04 b	16.63 ± 0.01 c	22.83 ± 0.05 b	25.99 ± 0.02 b
R150-100	14.68 ± 0.01 a	46.09 ± 0.03 c	16.74 ± 0.02 a	22.49 ± 0.01 d	25.84 ± 0.00 c
R180-100	14.36 ± 0.01 b	46.25 ± 0.06 a	16.80 ± 0.01 a	22.58 ± 0.06 c	25.89 ± 0.02 b
CK-100	14.26 ± 0.01 c	45.95 ± 0.03 d	16.68 ± 0.01 b	23.11 ± 0.03 a	26.11 ± 0.01 a

Note: Values are expressed as mean ± SD (*n* = 3). Values with different lowercase letters within the same column indicate significant differences among different roasting temperatures for the 100-mesh fraction (*p* < 0.05).

**Table 6:** Effects of roasting temperature on the digestibility fractions of starch samples.

Sample	RDS (%)	SDS (%)	RS (%)
CK-100	9.30 ± 0.04 a	14.37 ± 0.07 a	76.33 ± 0.04 d
R130-100	2.95 ± 0.11 c	9.19 ± 0.13 c	87.87 ± 0.02 b
R150-100	2.39 ± 0.01 d	8.26 ± 0.38 d	89.36 ± 0.39 a
R180-100	5.70 ± 0.37 b	11.81 ± 0.81 b	82.50 ± 0.42 c

Note: Values are expressed as mean ± SD (*n* = 3). Different lowercase letters within the same column indicate significant differences (*p* < 0.05). RDS: Rapidly Digestible Starch; SDS: Slowly Digestible Starch; RS: Resistant Starch.

## Data Availability

The original contributions presented in this study are included in the article. Further inquiries can be directed to the corresponding author.
